# Peripheral Non-Contrast MR Angiography Using FBI: Scan Time and T2 Blurring Reduction with 2D Parallel Imaging

**DOI:** 10.3390/jimaging10090223

**Published:** 2024-09-09

**Authors:** Won C. Bae, Lewis Hahn, Vadim Malis, Anya Mesa, Diana Vucevic, Mitsue Miyazaki

**Affiliations:** 1 Department of Radiology, University of California-San Diego, La Jolla, CA 92093, USA; wbae@health.ucsd.edu (W.C.B.);; 2 Department of Radiology, VA San Diego Healthcare System, San Diego, CA 92161, USA

**Keywords:** non-contrast MR angiography, fresh blood imaging (FBI), 2D parallel imaging, peripheral artery disease, atherosclerosis, SPEEDER

## Abstract

Non-contrast magnetic resonance angiography (NC-MRA), including fresh blood imaging (FBI), is a suitable choice for evaluating patients with peripheral artery disease (PAD). We evaluated standard FBI (sFBI) and centric ky-kz FBI (cFBI) acquisitions, using 1D and 2D parallel imaging factors (PIFs) to assess the trade-off between scan time and image quality due to blurring. The bilateral legs of four volunteers (mean age 33 years, two females) were imaged in the coronal plane using a body array coil with a posterior spine coil. Two types of sFBI and cFBI sequences with 1D PIF factor 5 in the phase encode (PE) direction (in-plane) and 2D PIF 3 (PE) × 2 (slice encode (SE)) (in-plane, through-slice) were studied. Image quality was evaluated by a radiologist, the vessel’s signal-to-noise ratio (SNR) and contrast-to-noise ratio (CNR) were measured, and major vessel width was measured on the coronal maximum intensity projection (MIP) and 80-degree MIP. Results showed significant time reductions from 184 to 206 s on average when using sFBI down to 98 to 162 s when using cFBI (*p* = 0.003). Similar SNRs (averaging 200 to 370 across all sequences and PIF) and CNRs (averaging 190 to 360) for all techniques (*p* > 0.08) were found. There was no significant difference in the image quality (averaging 4.0 to 4.5; *p* > 0.2) or vessel width (averaging 4.1 to 4.9 mm; *p* > 0.1) on coronal MIP due to sequence or PIF. However, vessel width measured using 80-degree MIP demonstrated a significantly wider vessel in cFBI (5.6 to 6.8 mm) compared to sFBI (4.5 to 4.7 mm) (*p* = 0.022), and in 1D (4.7 to 6.8 mm) compared to 2D (4.5 to 5.6 mm) (*p* < 0.05) PIF. This demonstrated a trade-off in T2 blurring between 1D and 2D PIF: 1D using a PIF of 5 shortened the acquisition window, resulting in sharper arterial blood vessels in coronal images but significant blur in the 80-degree MIP. Two-dimensional PIF for cFBI provided a good balance between shorter scan time (relative to sFBI) and good sharpness in both in- and through-plane, while no benefit of 2D PIF was seen for sFBI. In conclusion, this study demonstrated the usefulness of FBI-based techniques for peripheral artery imaging and underscored the need to strike a balance between scan time and image quality in different planes through the use of 2D parallel imaging.

## 1. Introduction

Peripheral artery disease (PAD) is an atherosclerotic disease affecting over 10 million Americans [1] that causes narrowing and blockage of the lumen of blood vessels via plaque buildup. Early detection of PAD can help prevent negative impacts on quality of life, including infection, limb loss, and death [1]. There are several non-invasive imaging methodologies for the diagnosis of PAD, each with limitations. Computed tomography angiography (CTA) is a popular modality that provides high-resolution diagnostic images. However, there are safety concerns, including long-term radiation exposure and high doses of contrast agents [2,3,4], which are undesirable for repeated imaging of diabetic patients with impaired kidney function [5]. Contrast-enhanced magnetic resonance angiography (CE-MRA) [6,7,8] provides a high signal-to-noise ratio (SNR), but the use of Gadolinium (Gd)-based contrast agents (GBCA) has been associated with nephrogenic systemic fibrosis (NSF) [9] and long-term deposition in the brain and other tissues [5,10,11,12]. Lastly, non-contrast MRA (NC-MRA) [13,14,15,16,17,18,19] does not pose these problems. 

**Figure 1:**
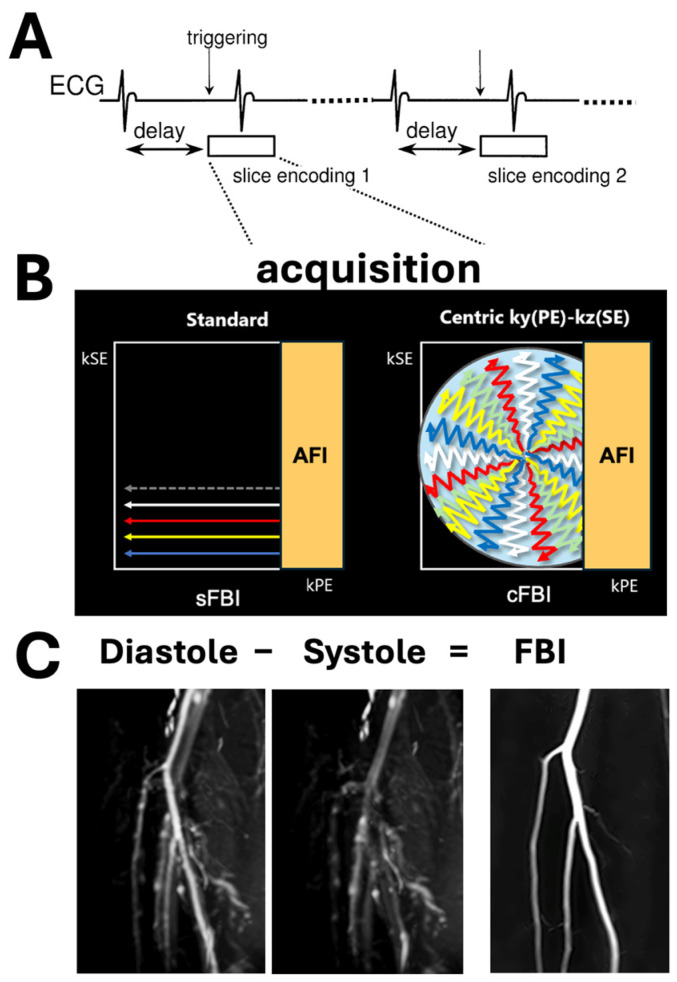
(**A**) Sequence diagram of electrocardiogram (ECG)-synchronized 3D half-Fourier fast spin echo (FSE). The 3D half-Fourier FSE sequence is ECG-synchronized for each slice encoding to obtain the same cardiac phase in every slice partition. (**B**) We utilized two different acquisition sequences of standard FBI (sFBI) and zigzag centric ky-kz trajectory FBI (cFBI), both with asymmetric Fourier imaging (AFI) in the phase encode direction. (**C**) FBI subtraction image (from Subject #1) is created from the subtraction of diastolic minus systolic source images.

Among the NC-MRA techniques available, fresh blood imaging (FBI) [13,14] and quiescent-interval single shot (QISS) [15] are widely used. QISS is a fast and simple two-dimensional (2D) NC-MRA method that acquires axial slices using a balanced steady-state free precession (bSSFP) [20] read-out perpendicular to the vessel orientation, similar to time-of-flight (TOF). FBI utilizes the blood signal difference between systole and diastole using an electrocardiographic (ECG)-gated 3D single-shot fast spin-echo (SSFSE) pulse sequence [13,14], illustrated in Figure 1A,B, in conjunction with image subtraction (Figure 1C). Regarding the zigzag centric ky-kz trajectory in Figure 1B (right), data are collected in the PE (ky) and SE (kz) directions [21,22]. 

**Figure 2:**
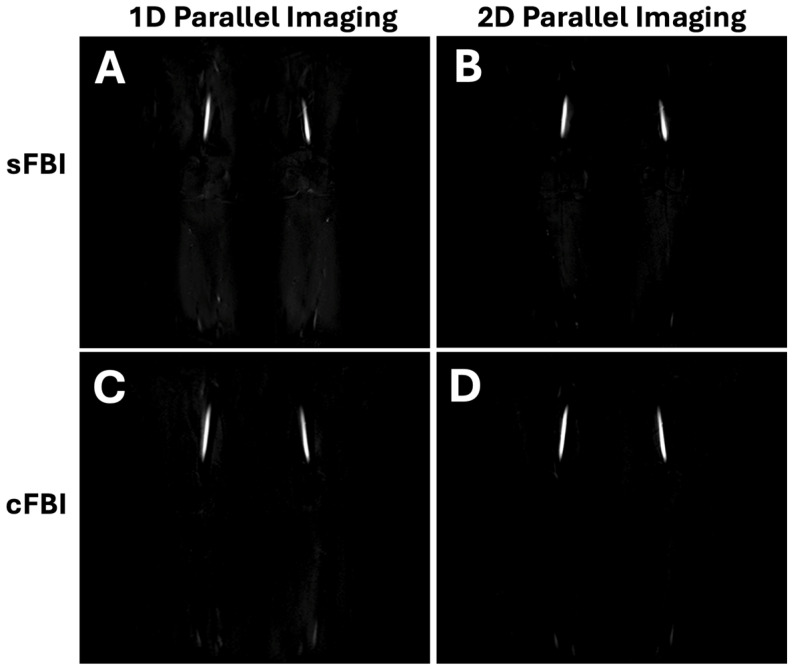
Subtraction (**A**,**B**) sFBI and (**C**,**D**) cFBI images acquired in the coronal plane using (**A**,**C**) 1D and (**B**,**D**) 2D parallel imaging. The images are from Subject #2.

During systole, images with a dark signal (flow void) in arteries with fast-flowing blood is acquired, while the slower blood flow during diastole generates a high arterial signal. The subtraction image depicts arteries with high signal intensity in the final FBI image (Figure 2A,B). The venous flow is relatively constant in both instances; thus, the venous contamination is effectively removed during subtraction. For clinical evaluation, maximum intensity projection (MIP) in the coronal plane is used to observe stenosis (Figure 2C,D) [23,24].

Due to the requirement of two (systolic and diastolic) acquisitions and multiple R-wave intervals, the standard FBI technique is somewhat slow, taking 3 to 6 min per station depending on the slab coverage (or number of slices), the number of shots to fill the k-space, and the subject’s heart rate (HR). Typical multi-station run-off imaging may take >10 min. Due to this, 3D partial-Fourier single- or few-shot acquisition using a standard k space (also known as standard FBI or sFBI) or zigzag centric ky-kz trajectories (centric FBI or cFBI), along with various parallel imaging schemes (or SPEEDER for Canon), are often used (Figure 1B). Since these sequences generally have a long echo train duration, parallel imaging is used to reduce the acquisition window. Increasing the parallel imaging factor (PIF) reduces the single-shot acquisition window, which reduces the T2 blurring effect [25,26,27]. Another way of reducing the acquisition window is increasing the number of shots to fill the k space at the expense of total acquisition time. Without applying PIF and/or increasing the number of shots, an acquisition window is relatively long, resulting in T2 blurring of the arterial blood with a T2 value of ~175 ms [28], unlike cerebrospinal fluid. In terms of the point spread function (PSF), arterial blood has a shorter T2 decay signal and a sharper decline in the modulation transfer function (MTF). This causes a wider and flatter curve in PSF compared to fluid signals [25].

For parallel imaging of peripheral arteries in the lower extremities, two parallel imaging coils of spine coils embedded in the bed and upper body coils are typically used, covering the top and bottom of the legs. PIF can be applied in both phase encoding (PE) and slice encoding (SE) directions. For sFBI, increasing the PIF in the PE direction alone may achieve a reduction in T2 blur, but for cFBI with a complex zigzag ky-kz trajectory into the SE direction (Figure 1B), PIF in both PE and SE directions may be beneficial. 

The purpose of this study was to demonstrate the T2 blurring of arterial blood vessels during non-contrast FBI imaging. We sought to determine the effect of (1) standard (sFBI) vs. centric FBI (cFBI) acquisition and (2) using 1D (in the phase encoding or PE direction only) vs. 2D (PE and slice encoding (SE) directions) PIFs on the scan time, image quality, image blurring, signal-to-noise (SNR), and contrast-to-noise ratios (CNR) when imaging peripheral arteries of the lower extremities in human subjects. 

## 2. Materials and Methods

Subjects: This human subject study was approved by the institutional review board. All procedures performed in this study involving human participants were compliant with the regulations of the Health Insurance Portability and Accountability Act (HIPAA). Healthy volunteers (*n* = 4, 2 females, 2 males, age range = 23 to 50 years old, mean age of 32.8 years old (standard deviation of 12.1 years old)) were recruited for this study. Inclusion criteria were healthy (no current pain or symptoms related to peripheral arteries) males and females aged between 18 and 60. Exclusion criteria included high BMI > 30 kg/m^2^, current or history of vascular diseases or surgery, and counter-indications for receiving MRI (such as claustrophobia, metal in the body, etc.). The age, sex, and resting heart rate of each subject are listed in Table 1.

MRI: Both legs of the volunteers were imaged at 3-T (Galan, Canon Medical Systems Corp., Otawara, Japan) using a 16-channel body coil covering the entire calf, in conjunction with a posterior 18-channel spine coil. Two FBI sequences (sFBI and cFBI) with different parallel imaging reduction factors (PIF) were used: (1) 1D PIF 5 (phase encode or PE direction of left–right) with an echo train length (ETL) of 40 and 69 for sFBI and cFBI, respectively, (2) 2D PIF 3 (PE direction of left–right) × 2 (slice encode or SE direction of anterior–posterior) for sFBI and cFBI using 2 shots with an echo train length of 64 and 61, respectively. Note that these ETL values are based on No Wrap of 1, which had to be increased in some thicker subjects to as high as 1.4 (this increased the ETL values). In all cases, we utilized the maximum PIF allowed by the manufacturer, although in theory, a greater PIF in the PE direction should be possible for the 2D parallel imaging. The remaining scan parameters were similar between the two sequences: single-shot (except for cFBI 2D parallel imaging used two-shot) fast spin echo (SSFSE) acquisition with read-out in the superior–inferior direction, TR = 2 R-R intervals or 1655 to 2250 ms (dependent on the HR), TE = 60 ms, FOV = 450 (PE) × 350 (RO) mm, matrix PE × RO = 320 × 320, flip angle/refocusing flip angles =90/180 deg, 45 × 2.6-mm slices, spectral attenuated inversion recovery (SPAIR) fat suppression, and number of averages = 1 for both systolic and diastolic acquisitions. Scan time varied from subject to subject due to differences in HR (Table 1). For each subject and the scan, the actual scan time was recorded to determine the mean and standard deviation (SD) in scan time between the two sequences.

Vessel Width: To determine the severity of blurring, we measured the cross-sectional width of the large right femoral artery on the coronal MIP to determine in-plane blurring and on 80-degree oblique MIP (close to the sagittal plane without overlapping the major arteries) to determine out-of-plane blurring in the anterior–posterior direction. ImageJ was used to draw a perpendicular line across the vessel and the full-width half maximum (FWHM) of the signal intensity was calculated and taken as the width of the vessel. 

Sharpness Grading: For qualitative assessment, coronal maximum intensity projection (MIP) images were reconstructed for all the series for side-by-side comparison. A board-certified radiologist with 6 years of experience performed visual grading of the sharpness of the vessel using a 1 to 5 scale that was more granular compared to similar existing schemes [29,30]: 1 = unacceptable, with severe noise and/or artifacts and impossible to interpret; 2 = poor, with moderate noise and/or artifacts and difficult to interpret; 3 = average, with mild noise and/or artifacts and interpretable; 4 = good, with nearly no noise and/or artifacts and easily interpretable; 5 = excellent. To determine inter-reader agreement, an additional reader, a researcher with over 20 years of experience in imaging of vascular diseases, performed the same reading. In cases of disagreement, the grade from the radiologist was used.

SNR, CNR: For quantitative assessment, a source image slice showing the largest cross-section of the femoral artery was selected. Regions of interest were placed in the left and right arteries, nearby muscles, and the background to determine the mean and standard deviation (SD) of the signal intensity (SI). The SNR of the artery was determined as the mean SI of the artery divided by the SD of the background. Similarly, the CNR of the artery was determined as the difference in the SNR between the artery and the muscle. 

Statistics: Effects of acquisition (sFBI vs. cFBI) and PIF (1D vs. 2D) on the mean scan time, SNR, CNR, vessel width, and visual grade were assessed using two-way repeated measures ANOVA, using JASP (Version 0.18.3) statistics software [31]. The significance level was set at *α* = 0.05. The power of the test (1-*β* error probability) was determined from the effect size for each factor, using the sum of squares values. G*Power software (Version 3.1.9.6) was used for this analysis [32,33]. Inter-reader agreement in grading was assessed using intraclass correlation analysis. 

## 3. Results

Scan Time (Table 2): There was a reduction in scan time through the use of both cFBI (*p* = 0.003) and 2D parallel imaging (*p* = 0.004). From sFBI 1D (scan time of 206 ± 38 s, mean ± standard deviation) to cFBI 1D (98 ± 15 s), there was an approximate time saving of 100 s, while from sFBI 1D to 2D PIF (184 ± 31 s), the time saved was about 20 s. However, from cFBI 1D (98 ± 15 s) to 2D (162 ± 38 s) parallel imaging, the mean scan time was increased by 65 s due to the use of two-shot acquisition in 2D.

**Figure 3:**
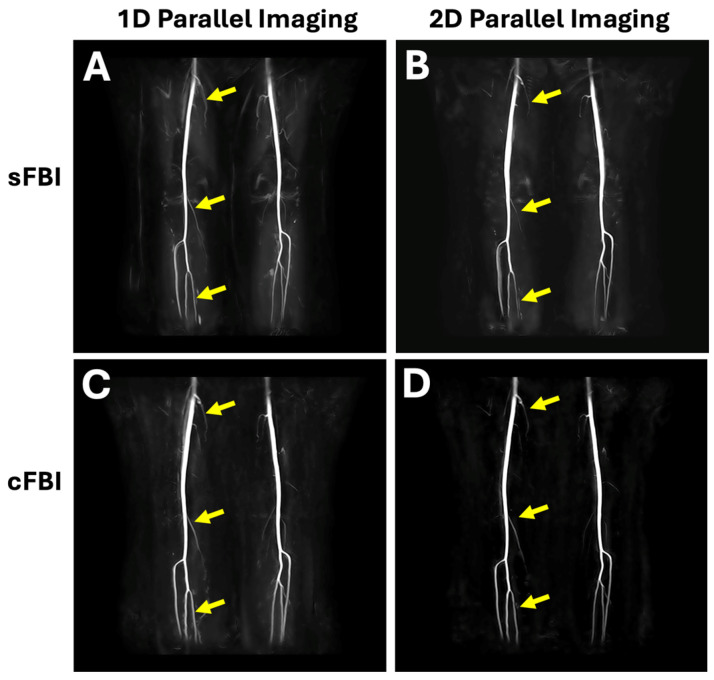
Coronal maximum intensity projection (MIP) images created from (**A**,**B**) sFBI and (**C**,**D**) cFBI subtraction images acquired using (**A**,**C**) 1D and (**B**,**D**) 2D parallel imaging. All of the images depicted the large peripheral artery with good contrast and sharpness. Using 1D parallel imaging factor or PIF (Figure 2C), smaller branched vessels (arrows) were more distinctly visible compared to those on the 2D PIF image (Figure 2D). Note that 1D PIF was applied with 5 (phase encode, PE) × 1 (slice encode, SE) and 2D PIF was applied with 3 (PE) × 2 (SE). The images are from Subject #2.

Observations: Visually, there was little difference in the overall quality and contrast of the subtraction FBI images (Figure 2), regardless of the acquisition technique. Coronal MIP images showed a slight but notable difference in the appearance of smaller vessels; using 1D PIF (Figure 3A,C), smaller branched vessels were more distinctly visible (arrows) compared to those on 2D PIF images (Figure 3B,D). Additionally, the femoral artery in sFBI with 2D PIF (Figure 3B) appeared slightly thicker than in other sequences. 

**Figure 4:**
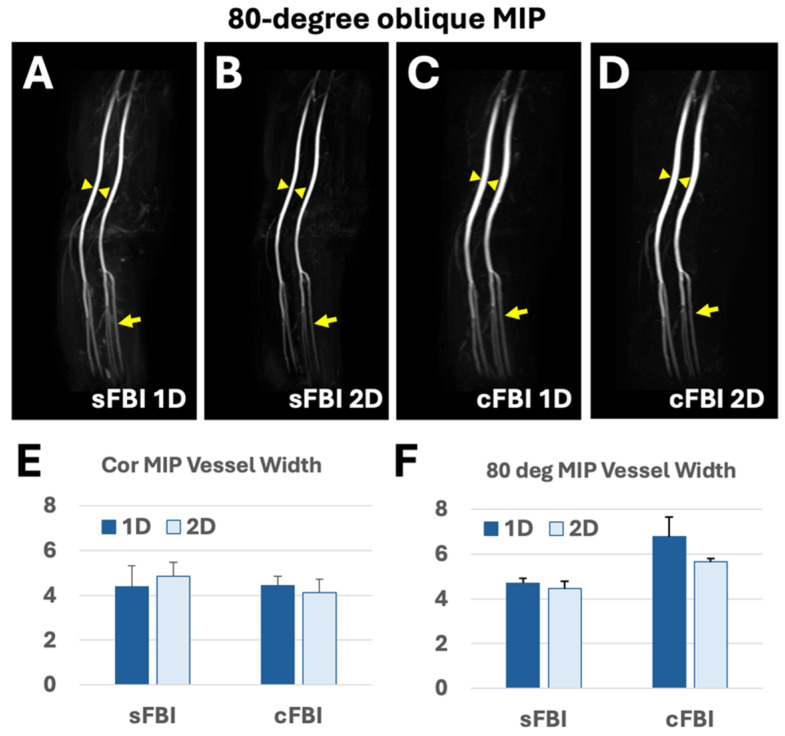
Oblique (nearly sagittal) maximum intensity projection or MIP images at 80-deg rotation, acquired with sFBI 1D parallel imaging factor or PIF (**A**), sFBI 2D PIF (**B**), cFBI 1D PIF (**C**), and cFBI 2D PIF (**D**). Arrowheads indicate measurement location for vessel width, and the arrows are pointed at smaller arteries. (**E**) Vessel widths measured on coronal MIP images showed no differences between techniques. *p*-value for acquisition (sFBI vs. cFBI) was 0.1, PIF (1D vs. 2D) was 0.5, and the interaction term was 0.053. Power for acquisition, PIF, and interaction were 0.45, 0.07, and 0.65, respectively. In contrast, (**F**) vessel widths measured on 80 deg oblique images demonstrated notably wider vessels (arrowheads) in cFBI compared to sFBI. *p*-value for acquisition was 0.022, PIF was 0.047, and the interaction term was 0.08. Power for acquisition, PIF, and interaction were 0.96, 0.16, and 0.10, respectively. For sFBI, the use of 2D PIF did not improve the 80 deg MIP image, which was expected since there is no acquisition difference in the SE direction when compared to 1D, but the blurring was slightly worse in the coronal MIP (**E**). Additionally, for cFBI, using 2D PIF further increased the width and caused blurring of smaller branching vessels (arrows). The images are from Subject #3.

In the 80-degree oblique coronal (nearly sagittal) MIP images (Figure 4), we assessed out-of-plane blurring in the anterior–posterior (AP) direction. Both of the sFBI images, 1D and 2D (Figure 4A,B), demonstrated no blurring in the AP direction. In contrast, both cFBI images exhibited a wider femoral artery (Figure 4C,D, arrowheads) compared in the sFBI images (Figure 4A,B).

Vessel Width: The observations corroborated with vessel width measurements. Vessel widths measured on coronal MIP (Figure 4E) were similar between techniques, averaging between 4.1 and 4.9 mm, without significant difference due to the sequence or 1D/2D PIF (each *p* > 0.05). In contrast, vessel width measured on 80-degree oblique images (Figure 4F and Table 3) demonstrated significantly wider (*p* = 0.022) vessels in cFBI compared to sFBI. Additionally, the use of 2D PIF resulted in a smaller width of the femoral artery (*p* = 0.047) compared to 1D PIF. The mean vessel width on sFBI was about 4.5 to 4.7 mm, while cFBI 1D had the highest mean width at 6.8 mm, followed by cFBI 2D at 5.6 mm.

**Figure 5:**
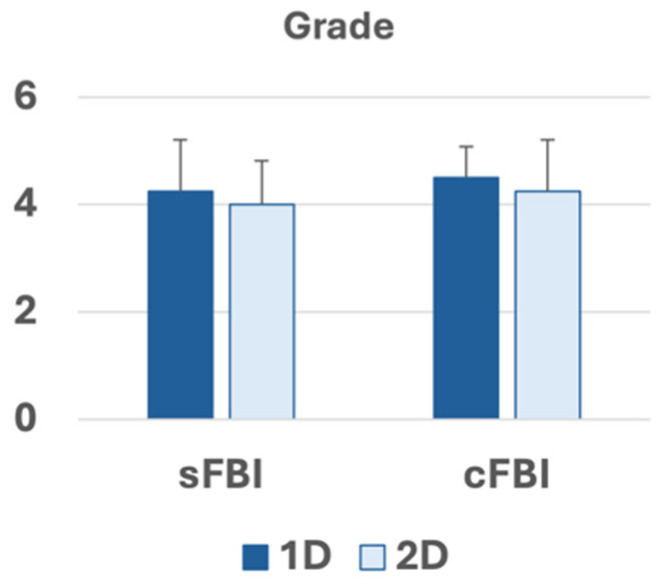
Grades of the sharpness of the vessels determined on coronal maximum intensity projection images. Mean +/− standard deviation, n = 4 subjects. *p*-value for acquisition (sFBI vs. cFBI) was 0.5, PIF (1D vs. 2D) was 0.2, and the interaction term was 1.0. Power for acquisition, PIF, and interaction were 0.56, 0.56, and 0.05, respectively.

Sharpness Grades: Figure 5 shows the visual grading of the sharpness of the vessel seen on coronal MIP images using a 1 to 5 scale. All of the images were graded 3 or higher, with an average grade above 4 for all techniques. We found no significant effect of the sequence (*p* = 0.5) or PIF (*p* = 0.2) on the grades. The inter-reader agreement was strong, with an intraclass correlation coefficient of 0.80.

**Figure 6:**
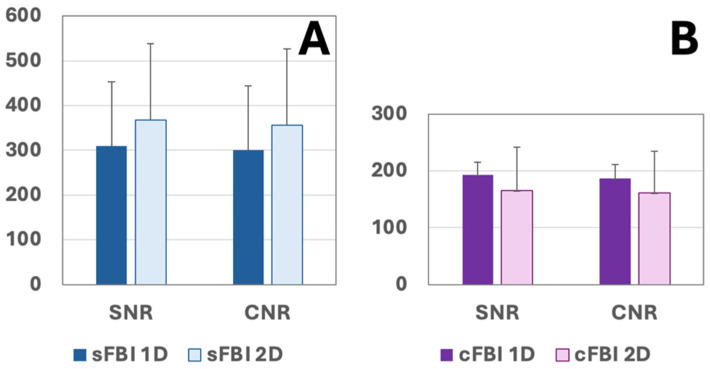
Signal-to-noise ratio (SNR) and contrast-to-noise ratio (CNR) of the femoral artery relative to surrounding muscle in (**A**) sFBI and (**B**) cFBI source images. Mean + standard deviation, n = 4 subjects. For SNR values, *p*-value for acquisition (sFBI vs. cFBI) was 0.3, PIF (1D vs. 2D) was 0.08, and the interaction term was 0.4. Power for acquisition, PIF, and interaction were 0.84, 0.32, and 0.06, respectively. For CNR values, *p*-value for acquisition was 0.3, PIF was 0.08, and the interaction term was 0.3. Power for acquisition, PIF, and interaction were 0.80, 0.34, and 0.06, respectively.

SNR, CNR: Both SNR and CNR values measured on the sFBI subtraction images were high, averaging over 300 regardless of the PIF (Figure 6A). While the mean SNR and CNR values were slightly higher for sFBI 2D, this was not statistically significant (*p* > 0.08). The results were similar for cFBI (Figure 6B). The mean SNR and CNR values were approximately 190 for cFBI 1D and approximately 270 for cFBI 2D. However, this was not statistically significant (*p* > 0.08). Between sFBI and cFBI, there were no statistical differences in SNR (*p* = 0.3) or CNR (*p* = 0.3).

## 4. Discussion

This study determined the effect of sFBI vs. cFBI, as well as using 1D vs. 2D PIF, by comparing scan time, image grade, SNR, CNR, and blurring in different planes when imaging the peripheral arteries of the lower extremities in healthy human subjects. We found a marked, greater than 50% time reduction from the use of cFBI and 1D PIF without the loss of image quality (by grading), SNR, or CNR. However, there was significant out-of-plane blurring in the anterior–posterior direction with cFBI, particularly when cFBI was combined with 1D PIF. This is due to the long acquisition window in the ky-kz direction. 

Balancing scan time and image quality is crucial in clinical practice. While shorter scan times enhance patient comfort and throughput, it must be done without compromising image quality, blurring, SNR, and CNR, which may impact diagnostic accuracy. Therefore, optimizing imaging protocols to achieve a balance between scan efficiency and image quality is paramount. All of the coronal MIP images were graded from good to excellent, with no statistical difference in the mean grade between the four techniques used. Although cFBI tended to have lower SNR and CNR values (~150 to 200) compared to sFBI (~300 to 350) measured in the subtraction images, this was not statistically significant. This has an important clinical implication: the existing sFBI protocol that takes 3 to 4 min may effectively be replaced with the cFBI protocol that takes 2–3 min, without sacrificing image quality and sharpness.

This study also revealed the trade-off in image quality and blurring when using 1D (PIF of 5 in the PE direction) vs. 2D (PIF of 3 (PE) × 2 (SE)) PIF. On 1D PIF coronal MIP images (Figure 3A,C), not only were major arteries rendered thin, but smaller branching vessels were also more distinctly visible compared to those on 2D PIF coronal MIP images (Figure 3B,D). However, this came at the trade-off of blurring in the slice direction for cFBI, as evidenced on 80-degree MIP images (Figure 4F): the 1D PIF cFBI 80 deg MIP image (Figure 4C) demonstrated thicker/more blurry vessels compared to the 2D PIF cFBI image (Figure 4D) when the vessel width was measured (Figure 4F). sFBI benefitted little from the use of 2D PIF in this regard. One-dimensional sFBI appeared sharper in coronal MIP than 2D sFBI, and both were similar in sharpness in oblique MIP. This was expected based on the known T2 blurring effect [26,27], where a longer acquisition window [27] and shorter T2 blood [25,26] result in greater blurring and signal loss due to the widening and flattening of the point spread function. Since cFBI is sampled in both PE and SE directions, the sequence benefitted from the use of 2D PIF in reducing T2 blur in the SE direction. In our study, the use of a high 1D PIF shortened the in-plane acquisition window, resulting in sharp coronal images for both sFBI and cFBI. However, for cFBI, substantial T2 blurring occurred in the slice direction. Two-dimensional PIF for cFBI remedied this and struck a better balance for sharpness in both the in-plane and slice directions, and this might be more appropriate if evaluation in different orientations (other than coronal MIP) is desired. cFBI 2D PIF may provide a great compromise between scan time (shorter than sFBI) and image quality/sharpness in this regard.

This study’s findings align with previous research highlighting the challenge of optimizing MRI protocols for vascular imaging. Compared to CE-MRA techniques, which can be acquired quickly in ~30 s or less [34], our cFBI is competitive but slightly slower. However, contrast-enhanced MRA is heavily dependent on the bolus timing, requiring consistent and reproducible contrast injection in each subject as well as fast acquisition. Poor technique may result in venous contamination and degrade the quality of the MRA. The FBI technique does not suffer from these limitations. Compared to non-contrast MRA, such as velocity-selective MRA (VS-MR) [35], our FBI techniques perform well. VS-MRA for the lower extremities requires a long scan time ranging from 5 to 8 min per station and has a somewhat lower artery-to-muscle CNR of around 30. However, it has demonstrated good diagnostic performance in the detection of peripheral artery stenosis, with an accuracy approaching 90% [35]. In one non-contrast hepatic MRA study that compared 1D (PIF factor 2) vs. 2D (PIF factor 2 × 2) for short tau inversion recovery acquisition, while a significant reduction in scan time was achieved with 2D PIF, it came at the expense of image degradation [36].

This early study has several limitations. First of all, our subjects were all healthy and relatively young, without any known vascular diseases. It is not clear whether vascular pathologies would change the outcome, for example, diagnostic performance may be affected by out-of-plane blurring in cFBI 1D. This must be investigated in future work that compares diagnostic performance in patients with peripheral artery diseases including diabetic patients. However, it is not unusual to perform sequence development in phantoms and healthy volunteers first, before transitioning to patients. Secondly, the number of subjects was small at only four; additional subjects are needed to ascertain if the current results hold and to make the results generalizable. Thirdly, due to the manufacturer-imposed limitation, we could not compare the same number of PIFs in the PE direction, i.e., comparing 5 (PE) × 1 (SE) vs. 5 (PE) × 2 (SE). Nonetheless, our results clearly demonstrate the effect of using 2D PIF on different sequences for MRA. Lastly, there may be additional confounders such as subject age and sex, which could be explored in detail in future studies involving a larger number of subjects.

## 5. Conclusions

In conclusion, this study demonstrated the usefulness of FBI-based techniques for peripheral artery imaging and underscored the need to strike a balance between scan time and image quality in different planes. The results demonstrated that a significant reduction in scan time while maintaining image quality is feasible, which could improve patient experience in clinical settings. Future studies involving a large number of PAD patients to validate and generalize the present findings will be needed. Ultimately, by understanding the implications of different techniques on image quality, diagnostic performance, and workflow efficiency, clinicians can tailor imaging protocols to optimize patient care effectively.

## Figures and Tables

**Table 1:** Subject characteristics. S.D.= standard deviation.

Subject #	Age (years)	Sex	HR (bpm)	BMI (kg/m^2^)
1	50	M	73	26.0
2	23	F	54	29.3
3	26	F	53	18.6
4	32	M	63	25.2
mean	32.8		60.8	24.8
S.D.	12.1		9.3	4.5

**Table 2:** Scan time of each technique in seconds. Mean (and standard deviation). *p*-value for acquisition (sFBI vs. cFBI) was 0.003, parallel imaging factor or PIF (1D vs. 2D) was 0.004, and the interaction term was 0.003. Power for acquisition, PIF, and interaction were 0.77, 0.10, and 0.30, respectively.

	1D PIF (s)	2D PIF (s)
sFBI	205.5 (37.7)	183.5 (30.9)
cFBI	97.8 (15.0)	162.0 (37.8)

**Table 3:** Femoral artery width in mm measured on an 80-degree oblique maximum intensity projection image. Mean (and standard deviation). *p*-value for acquisition (sFBI vs. cFBI) was 0.022, parallel imaging factor or PIF (1D vs. 2D) was 0.047, and the interaction term was 0.083. Power for acquisition, PIF, and interaction were 0.96, 0.16, and 0.10, respectively.

	1D PIF (mm)	2D PIF (mm)
sFBI	4.7 (0.22)	4.5 (0.33)
cFBI	6.8 (0.86)	5.6 (0.15)

## Data Availability

The data that support the findings of this study are not publicly available due to reasons of sensitivity. Anonymized data may be available from the corresponding author upon the review of the request. Data are located in controlled access data storage at the corresponding author’s institution.

## References

[1] Selvin E., Erlinger T.P. (2004). Prevalence of and risk factors for peripheral arterial disease in the United States: Results from the National Health and Nutrition Examination Survey, 1999–2000. Circulation.

[2] Iezzi R., Santoro M., Marano R., Di Stasi C., Dattesi R., Kirchin M., Tinelli G., Snider F., Bonomo L. (2012). Low-dose multidetector CT angiography in the evaluation of infrarenal aorta and peripheral arterial occlusive disease. Radiology.

[3] Nadolski G.J., Stavropoulos S.W. (2013). Contrast alternatives for iodinated contrast allergy and renal dysfunction: Options and limitations. J. Vasc. Surg..

[4] Fleischmann D., Hallett R.L., Rubin G.D. (2006). CT angiography of peripheral arterial disease. J. Vasc. Interv. Radiol..

[5] DeLoach S.S., Mohler E.R. (2007). Peripheral arterial disease: A guide for nephrologists. Clin. J. Am. Soc. Nephrol..

[6] Leung D.A., Debatin J.F. (1997). Three-dimensional contrast-enhanced magnetic resonance angiography of the thoracic vasculature. Eur. Radiol..

[7] Ho K.Y., Leiner T., de Haan M.W., Kessels A.G., Kitslaar P.J., van Engelshoven J.M. (1998). Peripheral vascular tree stenoses: Evaluation with moving-bed infusion-tracking MR angiography. Radiology.

[8] Lim R.P., Koktzoglou I. (2015). Noncontrast magnetic resonance angiography: Concepts and clinical applications. Radiol. Clin. N. Am..

[9] Aime S., Caravan P. (2009). Biodistribution of gadolinium-based contrast agents, including gadolinium deposition. J. Magn. Reson. Imaging.

[10] Kanda T., Ishii K., Kawaguchi H., Kitajima K., Takenaka D. (2014). High signal intensity in the dentate nucleus and globus pallidus on unenhanced T1-weighted MR images: Relationship with increasing cumulative dose of a gadolinium-based contrast material. Radiology.

[11] Kanda T., Osawa M., Oba H., Toyoda K., Kotoku J., Haruyama T., Takeshita K., Furui S. (2015). High Signal Intensity in Dentate Nucleus on Unenhanced T1-weighted MR Images: Association with Linear versus Macrocyclic Gadolinium Chelate Administration. Radiology.

[12] McDonald R.J., McDonald J.S., Kallmes D.F., Jentoft M.E., Murray D.L., Thielen K.R., Williamson E.E., Eckel L.J. (2015). Intracranial Gadolinium Deposition after Contrast-enhanced MR Imaging. Radiology.

[13] Miyazaki M., Sugiura S., Tateishi F., Wada H., Kassai Y., Abe H. (2000). Non-contrast-enhanced MR angiography using 3D ECG-synchronized half-Fourier fast spin echo. J. Magn. Reson. Imaging.

[14] Miyazaki M., Takai H., Sugiura S., Wada H., Kuwahara R., Urata J. (2003). Peripheral MR angiography: Separation of arteries from veins with flow-spoiled gradient pulses in electrocardiography-triggered three-dimensional half-Fourier fast spin-echo imaging. Radiology.

[15] Edelman R.R., Sheehan J.J., Dunkle E., Schindler N., Carr J., Koktzoglou I. (2010). Quiescent-interval single-shot unenhanced magnetic resonance angiography of peripheral vascular disease: Technical considerations and clinical feasibility. Magn. Reson. Med..

[16] Fan Z., Sheehan J., Bi X., Liu X., Carr J., Li D. (2009). 3D noncontrast MR angiography of the distal lower extremities using flow-sensitive dephasing (FSD)-prepared balanced SSFP. Magn. Reson. Med..

[17] Fan Z., Zhou X., Bi X., Dharmakumar R., Carr J.C., Li D. (2011). Determination of the optimal first-order gradient moment for flow-sensitive dephasing magnetization-prepared 3D noncontrast MR angiography. Magn. Reson. Med..

[18] Shin T., Hu B.S., Nishimura D.G. (2013). Off-resonance-robust velocity-selective magnetization preparation for non-contrast-enhanced peripheral MR angiography. Magn. Reson. Med..

[19] Shin T., Qin Q., Park J.Y., Crawford R.S., Rajagopalan S. (2016). Identification and reduction of image artifacts in non-contrast-enhanced velocity-selective peripheral angiography at 3T. Magn. Reson. Med..

[20] Wu G., Yang J., Zhang T., Morelli J.N., Giri S., Li X., Tang W. (2016). The diagnostic value of non-contrast enhanced quiescent interval single shot (QISS) magnetic resonance angiography at 3T for lower extremity peripheral arterial disease, in comparison to CT angiography. J. Cardiovasc. Magn. Reson..

[21] Malis V., Vucevic D., Bae W.C., Yamamoto A., Kassai Y., Lane J., Hsiao A., Nakamura K., Miyazaki M. Fast Non-Contrast MR Angiography using Zigzag Centric ky-kz k-space Trajectory and exponential refocusing flip angles with restoration of longitudinal magnetization. Magn. Reson. Med. Sci..

[22] Ota H., Morita Y., Vucevic D., Higuchi S., Takagi H., Kutsuna H., Yamashita Y., Kim P., Miyazaki M. (2024). Motion robust coronary MR angiography using zigzag centric ky-kz trajectory and high-resolution deep learning reconstruction. Magn. Reson. Mater. Phys. Biol. Med..

[23] Miyazaki M., Akahane M. (2012). Non-contrast enhanced MR angiography: Established techniques. J. Magn. Reson. Imaging.

[24] Miyazaki M., Lee V.S. (2008). Nonenhanced MR angiography. Radiology.

[25] Constable R.T., Gore J.C. (1992). The loss of small objects in variable TE imaging: Implications for FSE, RARE, and EPI. Magn. Reson. Med..

[26] Miyazaki M., Ichinose N., Sugiura S., Kassai Y., Kanazawa H., Machida Y. (1998). A novel MR angiography technique: SPEED acquisition using half-Fourier RARE. J. Magn. Reson. Imaging.

[27] Qin Q. (2012). Point spread functions of the T2 decay in k-space trajectories with long echo train. Magn. Reson. Imaging.

[28] Lu H., Xu F., Grgac K., Liu P., Qin Q., van Zijl P. (2012). Calibration and validation of TRUST MRI for the estimation of cerebral blood oxygenation. Magn. Reson. Med..

[29] Boujan T., Neuberger U., Pfaff J., Nagel S., Herweh C., Bendszus M., Mohlenbruch M.A. (2018). Value of Contrast-Enhanced MRA versus Time-of-Flight MRA in Acute Ischemic Stroke MRI. AJNR Am. J. Neuroradiol..

[30] Krishnam M.S., Tomasian A., Deshpande V., Tran L., Laub G., Finn J.P., Ruehm S.G. (2008). Noncontrast 3D steady-state free-precession magnetic resonance angiography of the whole chest using nonselective radiofrequency excitation over a large field of view: Comparison with single-phase 3D contrast-enhanced magnetic resonance angiography. Investig. Radiol..

[31] Team J. (2024). JASP.

[32] Faul F., Erdfelder E., Buchner A., Lang A.G. (2009). Statistical power analyses using G*Power 3.1: Tests for correlation and regression analyses. Behav. Res. Methods.

[33] Kang H. (2021). Sample size determination and power analysis using the G*Power software. J. Educ. Eval. Health Prof..

[34] Nielsen Y.W., Thomsen H.S. (2012). Contrast-enhanced peripheral MRA: Technique and contrast agents. Acta Radiol..

[35] Shin T., Menon R.G., Thomas R.B., Cavallo A.U., Sarkar R., Crawford R.S., Rajagopalan S. (2019). Unenhanced Velocity-Selective MR Angiography (VS-MRA): Initial Clinical Evaluation in Patients with Peripheral Artery Disease. J. Magn. Reson. Imaging.

[36] Shimada K., Isoda H., Okada T., Kamae T., Arizono S., Hirokawa Y., Shibata T., Togashi K. (2011). Non-contrast-enhanced hepatic MR angiography: Do two-dimensional parallel imaging and short tau inversion recovery methods shorten acquisition time without image quality deterioration?. Eur. J. Radiol..

